# Rationally designed ligand‐independent peptide inhibitors of TREM‐1 ameliorate collagen‐induced arthritis

**DOI:** 10.1111/jcmm.13173

**Published:** 2017-04-06

**Authors:** Zu T. Shen, Alexander B. Sigalov

**Affiliations:** ^1^ SignaBlok Inc Shrewsbury MA USA

**Keywords:** triggering receptor expressed on myeloid cells 1, macrophages, therapeutic peptides, signalling chain homo‐oligomerization model of cell signalling, targeted delivery, inflammation, collagen‐induced arthritis

## Abstract

Triggering receptor expressed on myeloid cells 1 (TREM‐1) is critically involved in the pathogenesis of rheumatoid arthritis (RA). In contrast to cytokine blockers, therapeutic blockade of TREM‐1 can blunt excessive inflammation while preserving the capacity for microbial control. However, the nature of the TREM‐1 ligand(s) and mechanisms of TREM‐1 signalling are still not yet well understood, impeding the development of clinically relevant inhibitors of TREM‐1. The aim of this study was to evaluate the anti‐arthritic activity of a novel, ligand‐independent TREM‐1 inhibitory nonapeptide GF9 that was rationally designed using the signalling chain homo oligomerization (SCHOOL) model of cell signalling. Free GF9 and GF9 bound to macrophage‐targeted nanoparticles that mimic human high‐density lipoproteins (GF9‐HDL) were used to treat collagen‐induced arthritis (CIA). We also tested if 31‐mer peptides with sequences from GF9 and helices 4 (GE31) and 6 (GA31) of the major HDL protein, apolipoprotein A‐I, are able to perform three functions: assist in the self‐assembly of GA/E31‐HDL, target these particles to macrophages and block TREM‐1 signalling. We showed that GF9, but not control peptide, ameliorated CIA and protected against bone and cartilage damage. The therapeutic effect of GF9 was accompanied by a reduction in the plasma levels of macrophage colony‐stimulating factor and pro‐inflammatory cytokines such as tumour necrosis factor‐α, interleukin (IL)‐1 and IL‐6. Incorporation of GF9 alone or as a part of GE31 and GA31 peptides into HDL significantly increased its therapeutic efficacy. Collectively, our findings suggest that TREM‐1 inhibitory SCHOOL sequences may be promising alternatives for the treatment of RA.

## Introduction

Macrophages are central to the pathogenesis of rheumatoid arthritis (RA) 1, an autoimmune disease that affects approximately 1% of the world population 2. The abundance and activation of macrophages in the inflamed synovial membrane significantly correlates with the severity of RA 1, 3. Therapies that fail to reduce the number of synovial sublining macrophages are unlikely to be clinically effective 4. Major targets related to macrophage development, activation, growth and differentiation include tumour necrosis factor‐α (TNFα), interleukin‐1 (IL‐1), IL‐6 and macrophage colony‐stimulating factor (M‐CSF or CSF‐1) 5. M‐CSF‐dependent cells are known to be essential for collagen‐induced arthritis (CIA) development 6. Targeted delivery of antirheumatic drugs to macrophages is another highly desirable strategy for the treatment of RA because it would not only strike the cells that mediate or amplify most of the permanent tissue destruction but also spare other cells that do not affect joint damage 7, 8.

**Figure 1:**
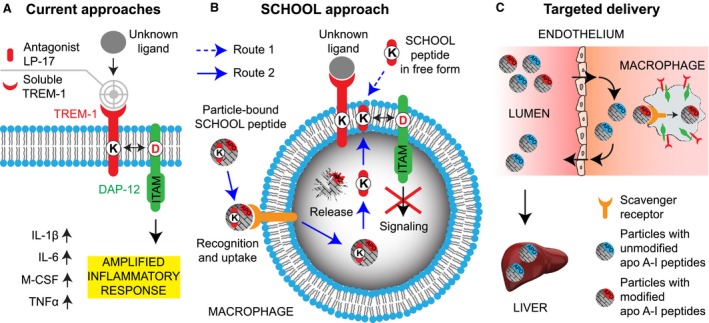
Schematic depiction for the proposed concepts of TREM‐1 inhibition and macrophage‐targeted drug delivery. **(A)** Current approaches to silencing TREM‐1 include the use of an antagonist LP‐17 and a soluble TREM‐1 receptor decoy. **(B)** As opposed to current approaches, synthetic peptides designed utilizing a novel model of immune signalling, the SCHOOL model, employ ligand‐independent mechanisms of action and block transmembrane interactions between TREM‐1 and its signalling partner DAP‐12. The peptides can be used in either free or particle‐bound form and reach their site of action from both outside or inside the cell (routes 1 and 2, respectively). **(C)** Synthetic HDL containing either methionine‐sulfoxidized apolipoprotein (apo) A‐I protein, the major HDL protein, or its peptides (depicted red) are uptaken by macrophages, thereby delivering any incorporated payload(s) to these cells. In contrast, native HDL with unmodified apo A‐I (depicted blue) transport excess cholesterol from peripheral tissues to the liver and are not normally uptaken by macrophages. Abbreviations: apo, apolipoprotein; DAP‐12, DNAX activation protein of 12 kDA; HDL, high‐density lipoproteins; IL, interleukin; ITAM, immunoreceptor tyrosine‐based activation motif; M‐CSF, macrophage colony‐stimulating factor; SCHOOL, signalling chain homooligomerization; TNF, tumour necrosis factor; TREM‐1, triggering receptor expressed on myeloid cells 1.

Triggering receptor expressed on myeloid cells 1 (TREM‐1) contributes to RA and is highly expressed in the synovium of RA patients 9. TREM‐1 activation enhances the release of TNFα, IL‐1β, IL‐6 and M‐CSF 9, 10. Blockade of TREM‐1 significantly ameliorates CIA 11. Importantly, recent studies in TREM‐1 knockout (KO) mice suggest that in contrast to cytokine blockers 12, therapeutic blockade of TREM‐1 can blunt excessive inflammation while preserving the capacity for microbial control 13. Further, human beings lacking DAP‐12, a signalling partner for TREM‐1 (Fig. 1A), do not have problems resolving infections 14. Together, this implicates TREM‐1 as a promising target for the development of new rational RA therapies.

Conventional TREM‐1 inhibitors such as a soluble TREM‐1 decoy receptor or a TREM‐1 antagonist LP‐17 block the receptor binding to its ligand (Fig. 1A). However, despite some recent evidence that peptidoglycan recognition protein 1 (PGLYRP1) may potentially act as a ligand for TREM‐1 15, the actual nature of the TREM‐1 ligand(s) and mechanisms of TREM‐1 signalling are still not yet well understood, impeding the development of clinically relevant inhibitors of TREM‐1. Recently, using a new model of transmembrane signalling, the signalling chain homooligomerization (SCHOOL) model 16, 17, we designed a TREM‐1‐specific inhibitory nonapeptide GF9 that employs a novel, ligand‐independent mechanism of receptor inhibition (Fig. 1B) 18. We demonstrated that GF9 attenuates the specific inflammatory response and ameliorates sepsis and non‐small cell lung cancer (NSCLC) in animal models 18. We also used self‐assembling lipopeptide complexes that mimic human high‐density lipoproteins (HDL) for targeted delivery of GF9 to macrophages (Fig. 1C) and showed that this increases its therapeutic efficacy *in vivo* 18.

In the present study, we investigated the therapeutic effects of free and HDL‐bound GF9 in CIA and demonstrated that GF9, but not control peptide GF9‐G, suppresses release of pro‐inflammatory cytokines and M‐CSF, decreases inflammation and protects against bone and cartilage destruction in CIA. We also investigated interactions of free and HDL‐bound GF9 with macrophages *in vitro* and showed that GF9 colocalizes with TREM‐1 in the cell membrane and can reach its site of action from both outside and inside the cell. We next designed peptides GE31 and GA31 with sequences from GF9 and helices 4 and 6 of the major HDL protein, apolipoprotein (apo) A‐I, respectively. We suggested that by combining these sequences, GA31 and GE31 will be able to perform three functions: assist in the self‐assembly of HDL, target HDL to macrophages and silence the TREM‐1 signalling pathway. We demonstrated, for the first time, that similar to GF9‐HDL, these lipopeptide complexes ameliorate CIA. Collectively, our findings suggest that TREM‐1 inhibitory SCHOOL sequences may be promising alternatives for the treatment of RA.

## Materials and Methods

### Chemicals, lipids and cells

Sodium cholate, cholesteryl oleate and other chemicals were purchased from Sigma‐Aldrich Company (St. Louis, MO, USA). 1,2‐dimyristoyl‐*sn*‐glycero‐3‐phosphocholine (DMPC), 1,2‐dimyristoyl‐*sn*‐glycero‐3‐phospho‐(1′‐rac‐glycerol) (DMPG), 1‐palmitoyl‐2‐oleoyl‐sn‐glycero‐3‐phosphocholine (POPC), 1‐palmitoyl‐2‐oleoyl‐*sn*‐glycero‐3‐phospho‐(1′‐rac‐glycerol) (POPG), 1,2‐dimyristoyl‐*sn*‐glycero‐3‐phosphoethanolamine‐N‐(lissamine rhodamine B sulfonyl) (Rho B‐PE) and cholesterol were purchased from Avanti Polar Lipids (Alabaster, AL, USA). The murine macrophage cell line J774A.1 was obtained from the American Type Culture Collection (ATCC, Manassas, VA, USA).

### Peptide synthesis

The following synthetic peptides were ordered from American Peptide Company (Sunnyvale, CA, USA): two 9‐mer peptides GFLSKSLVF (human TREM‐1_213‐221_, GF9) and GFLSGSLVF (GF9‐G), two 22‐mer methionine‐sulfoxidized peptides PYLDDFQKKWQEEM(O)ELYRQKVE (H4) and PLGEEM(O)RDRARAHVDALRTHLA (H6) that correspond to human apo A‐I helices 4 (apo A‐I_123‐144_) and 6 (apo A‐I_167‐188_), respectively, and two 31‐mer methionine‐sulfoxidized peptides, GFLSKSLVFPYLDDFQKKWQEEM(O)ELYRQKVE (GE31) and GFLSKSLVFPLGEEM(O)RDRARAHVDALRTHLA (GA31). All peptides were purified by reversed‐phase high‐performance liquid chromatography (RP‐HPLC), and their purity was confirmed by amino acid analysis and mass spectrometry.

### Fluorescent labelling of peptides

Oxidized apo A‐I H4 peptide was solubilized using 0.1 M phosphate, pH 8, reacted with two‐fold molar excess of DyLight 488 N‐hydroxysuccinimide (NHS) ester and incubated at 25°C for 3 hr. GF9 peptide was solubilized using 0.07 M sodium bicarbonate, pH 9 and 85% dimethyl sulfoxide (v/v), reacted with 1.5‐fold molar excess of DyLight 488 NHS ester and incubated at 25°C for 5 hr. All reactions were quenched using ethanolamine. The reaction mixture was purified using RP‐HPLC.

### Lipoproteins

Discoidal HDL (dHDL) complexes that contain GF9 (GF9‐dHDL) or an equimolar mixture of GA31 and GE31 (GA/E31‐dHDL) were synthesized essentially as previously described 18. The molar ratio was 65:25:3:1:190 corresponding to POPC:POPG:GF9:apo A‐I:sodium cholate for GF9‐dHDL that contain GF9 and an equimolar mixture of oxidized apo A‐I peptides H4 and H6 peptides or 65:25:1:190 corresponding to DMPC:DMPG:GA/E31:sodium cholate for GA/E31‐dHDL that contain an equimolar mixture of oxidized peptides GA31 and GE31. In subsets of experiments, GF9 or apo A‐I H4 were DyLight 488‐labelled. Spherical HDL (sHDL) complexes that contain GF9 (GF9‐sHDL) or an equimolar mixture of GA31 and GE31 (GA/E31‐sHDL) were synthesized using the sodium cholate dialysis procedure substantially as previously described 18, 19. The molar ratio was 125:6:2:3:1:210 corresponding to POPC:cholesterol:cholesteryl oleate:GF9:apo A‐I:sodium cholate for GF9‐sHDL that contain GF9 and an equimolar mixture of oxidized apo A‐I peptides H4 and H6 or 125:6:2:1:210 corresponding to POPC:cholesterol:cholesteryl oleate:GA/E31‐I:sodium cholate for GA/E31‐sHDL that contain an equimolar mixture of oxidized peptides GA31 and GE31. In subsets of experiments, GF9 or apo A‐I H4 was DyLight 488‐labelled. All obtained HDL formulations were purified and characterized as described previously 18, 19, 20.

### Confocal analysis

BALB/c murine macrophage J774A.1 cells were grown at 37°C in six‐well tissue culture plates containing glass coverslips. After reaching target confluency of approximately 50%, cells were incubated for 6 h at 37°C with Rho B‐labelled HDL. In subsets of experiments, Rho B‐labelled HDL that contain DyLight 488‐labelled apo A‐I H4 or DyLight 488‐labelled GF9 were used. TREM‐1 staining was performed as described 21. ProLong Gold Antifade DAPI (4′,6‐diamidino‐2‐phenylindole) mounting medium was used to mount coverslips, and the slides were photographed using an Olympus BX60 fluorescence microscope. Confocal imaging was performed with a Leica TCS SP5 II laser scanning confocal microscope as previously reported 19, 22.

### Ethics statements

All animal experiments were performed in strict accordance with the recommendations in the Guide for the Care and Use of Laboratory Animals of the National Institutes of Health (NIH) and in the United States Department of Agriculture (USDA) Animal Welfare Act (9 CFR, Parts 1, 2, and 3). All experimental procedures were approved by the Institutional Animal Care and Use Committee of Bolder BioPATH for compliance with regulations prior to study initiation (Animal Welfare Assurance number A7649‐06), and all experiments were performed in accordance with the approved protocol BBP‐001.B.

### Collagen‐induced arthritis (CIA) model

Animal studies were performed by Bolder BioPATH (Boulder, CO, USA). CIA was induced in male 6‐ to 7‐week‐old DBA/1 mice by immunization with bovine type II collagen as previously described 19. Briefly, mice were injected intradermally with 100 μl of Freund's complete adjuvant containing 250 μg of bovine type II collagen (2 mg/ml final concentration) at the base of the tail on day 0 and again on day 21. On day 24, mice were randomized by body weight into treatment groups. At enrolment on day 24, the mean mouse weight was 20 g. Arthritis onset occurred on days 26‐38. Starting day 24, mice were injected intraperitoneally (i.p.) daily for 14 consecutive days with GF9 (2.5 and 25 mg/kg), GF9‐G (25 mg/kg), GF9‐dHDL (2.5 mg/kg), GF9‐sHDL (2.5 mg/kg), GA/E31‐dHDL (dose equivalent to 4 mg of GF9/kg), GA/E31‐sHDL (dose equivalent to 4 mg of GF9/kg) or with PBS. Mice were weighed on study days 24, 26, 28, 30, 32, 34, 36 and 38 (prior to necropsy). Daily clinical scores were given on a scale of 0–5 for each of the paws on days 24–38 using previously described criteria 19. On day 38, mice were killed for necropsy.

### Histology assessment of joints

At the end of study, fore paws, hind paws and knees were harvested, fixed in 10% neutral buffered formalin for 1–2 days, and then decalcified in 5% formic acid for 4–5 days before standard processing for paraffin embedding. Sections (8 μm) were cut and stained with toluidine blue (T blue). Hind paws, fore paws and knees were embedded and sectioned in the frontal plane. Six joints from each animal were processed for histopathological evaluation. The joints were then assessed using 0–5 scale for inflammation, pannus formation, cartilage damage, bone resorption and periosteal new bone formation as previously reported 19. A summed histopathology score (sum of five parameters, 0–25 scale) was also determined.

### Cytokine detection

Plasma was collected on days 24, 30 and 38, and cytokines were analysed by Quantibody Mouse Cytokine Array Q1 kits (RayBiotech, Norcross, GA, USA) according to the manufacturer's instructions.

### Statistical analysis

All statistical analyses were performed with GraphPad Prism 6.0 software (GraphPad, La Jolla, CA, USA). Results are expressed as the mean ± SEM. Statistical differences were analysed using analysis of variance with Bonferroni adjustment. *P* values less than 0.05 were considered significant.

### Sequence accession numbers

Accession numbers (UniProtKB/Swiss‐Prot knowledgebase, http://www.uniprot.org/) for the protein sequences discussed in this Research Article is as the follows: human TREM‐1, Q9NP99; human apo A‐I, P02647.

## Results

### Intracellular uptake of GF9‐HDL by macrophages and colocalization of GF9 with TREM‐1

**Figure 2:**
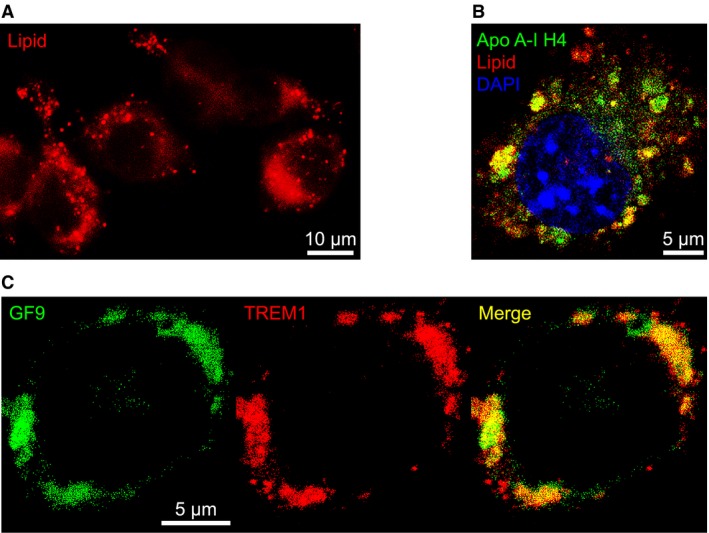
Interaction of GF9‐loaded high‐density lipoproteins (HDL) with macrophages and colocalization of GF9 with TREM‐1. **(A)** Fluorescence microscopy reveals a punctuated pattern of the interaction between GF9‐loaded spherical HDL (GF9‐sHDL) and J774A.1 macrophages that closely mimics that of the receptor‐mediated physiological interaction between native HDL and hepatocytes. Cells were incubated for 6 h at 37°C with GF9‐sHDL that contain rhodamine (Rho) B‐labelled peptide (red). Scale bar = 10 μm. **(B)** Confocal microscopy demonstrates that upon interaction, both lipid and protein components of GF9‐sHDL are delivered intracellularly to macrophages. Cells were incubated for 6 h at 37°C with GF9‐sHDL that contain Rho B‐labelled lipid (red) and DyLight 488‐labelled apo A‐I peptide H4 (green). Cell nuclei were stained with 4′,6‐diamino‐2‐phenylindole (DAPI) dye (blue). Scale bar = 5 μm. **(C)** Colocalization of GF9 and TREM‐1 in the cell membrane. Cells were incubated for 6 h at 37°C with GF9‐sHDL that contain DyLight 488‐labelled GF9 (green) and stained for TREM‐1 with Alexa 647‐labelled anti‐TREM‐1 antibody (red). Scale bar = 5 μm.

Previously, we reported that oxidation of apo A‐I or its peptides H4 and H6 significantly enhances *in vitro* macrophage uptake of GF9‐HDL 18. In this study, using fluorescence microscopy and GF9‐HDL with Rho B‐labelled lipid, we first demonstrated a punctuated pattern of the interaction between GF9‐HDL and macrophages (Fig. 2A), which closely mimics that of the physiological interaction between native HDL and hepatocytes, which is mediated by scavenger receptor BI (SR‐BI) 23. To confirm intracellular uptake *versus* nonspecific cell surface binding, we next examined the interaction between J774 macrophages and GF9‐HDL that contain Rho B‐labelled lipid and DyLight 488‐labelled oxidized apo A‐I peptide H4. This interaction resulted in intracellular delivery of both lipid and peptide components of GF9‐HDL (Fig. 2B), suggesting that the whole GF9‐HDL particle is uptaken by the cell, most likely by a receptor‐mediated mechanism. Pronounced colocalization of lipid and apo A‐I peptide H4 (Fig. 2B) demonstrates that at this time‐point, most of the GF9‐HDL particles remain intact after uptake, when the others are degraded, releasing their lipid and peptide contents into the intracellular space. While the data illustrated in Figure 2A and B were generated using GF9‐sHDL, similar results were observed for GF9‐dHDL (data not shown). Our data also indicate that the use of an equimolar mixture of oxidized peptides GA31 and GE31 enhances uptake of GA/E31‐HDL of discoidal and spherical shape by macrophages *in vitro* as compared with their unmodified counterparts (data not shown). Together, these findings suggest that oxidized apo A‐I epitopes responsible for the interaction with macrophages are exposed in all types of the HDL particles used.

T cell receptor (TCR) SCHOOL peptide, known as TCR core peptide 21, colocalizes with TCR in the T cell membrane 21. Recently, we demonstrated that another TCR SCHOOL peptide MG11, the peptide derived from the severe acute respiratory syndrome coronavirus fusion peptide sequence, colocalizes with TCR and exhibits predicted T cell‐related therapeutic activity in CIA 19, 24. In the present study, we show that GF9 self‐inserts into the cell membrane and colocalizes with TREM‐1 (Fig. 2C). Importantly, colocalization of GF9 with TREM‐1 is observed after incubation of macrophages with either free GF9 (data not shown) or sHDL‐bound GF9 (Fig. 2C). This indicates that GF9 can reach its intramembrane site of action from both outside and inside the cell.

Thus, the use of HDL‐based platform for macrophage‐targeted delivery results in intracellular uptake followed by release of the incorporated GF9 and its self‐insertion into the cell membrane from inside the cell (Fig. 1B, Route 2). Further, as suggested, oxidized GA31 and GE31 not only assist in the self‐assembly of HDL but also target these particles to macrophages.

### Reduction of inflammation and suppression of the clinical severity of CIA

To evaluate an anti‐arthritic activity of free and HDL‐bound TREM‐1 inhibitory GF9 sequences that were designed using our concepts of transmembrane signalling 16, 17, 18 and macrophage‐targeted drug delivery 18, 22, 25, we utilized the CIA mouse model, the most widely studied autoimmune model of RA 26.

**Figure 3:**
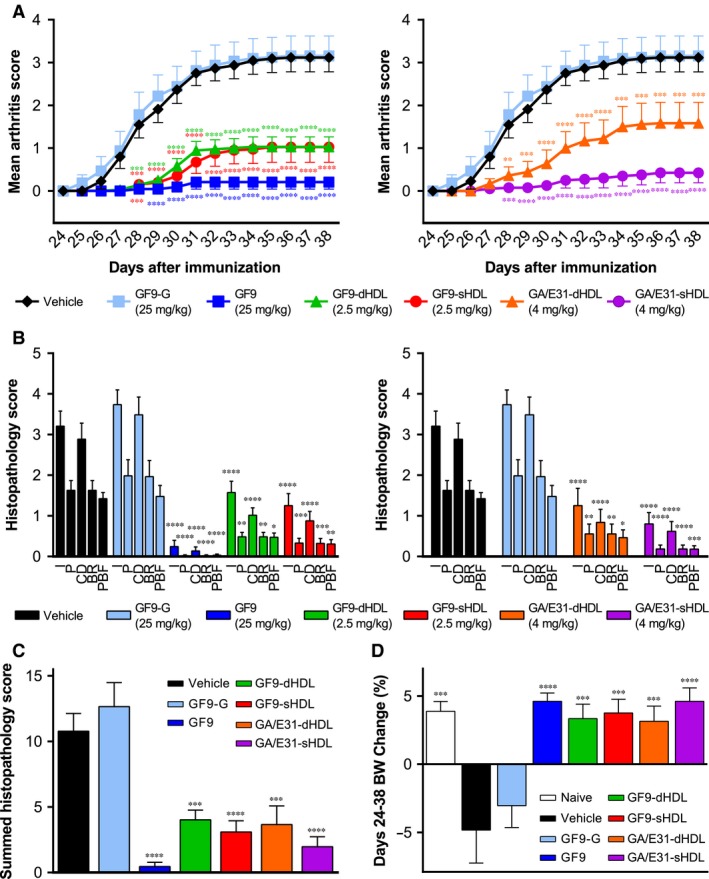
Treatment with TREM‐1 inhibitory GF9 sequences ameliorates collagen‐induced arthritis (CIA). As described in the ‘Materials and Methods’, starting on day 24 after immunization, mice with CIA were intraperitoneally (i.p.) administered daily for 14 consecutive days with vehicle (black), GF9‐G (light blue), GF9 (dark blue), GF9‐loaded discoidal high‐density lipoproteins (GF9‐dHDL, green), GF9‐loaded spherical HDL (GF9‐sHDL, red), and d‐ or sHDL that contain an equimolar mixture of trifunctional peptides GA31 and GE31 (GA/E31‐dHDL, orange, and GA/E31‐sHDL, purple, respectively). **(A)** Clinical arthritis score. **(B** and **C)** On day 38, mice were killed and the histopathological examination of mouse joints was performed. Histopathological scores of inflammation (I), pannus (P), cartilage damage (CD), bone resorption (BR) and periosteal new bone formation (PBF) are shown in (**B**). Summed histopathology scores calculated as the sum of all five histopathological parameters are shown in (**C**). **(D)** Mouse body weight (BW) was measured every other day from day 24 to day 38. Mean BW changes were calculated as a percentage of the difference between beginning (day 24) and final (day 38) BW. All results are expressed as the mean ± SEM (n = 10 mice per group). *P <* *0.05; **P <* *0.01; ***P <* *0.001; and ****P <* *0.0001 *versus* vehicle.

When administered at 25 mg/kg, GF9 but not a control peptide GF9‐G significantly suppressed arthritis severity when compared with administration of vehicle. The difference between the GF9 and GF9‐G groups started on day 28 and continued until day 38 (Fig. 3A). On day 38, the mean ± SEM clinical arthritis score in mice treated with GF9 was much lower than that observed in GF9‐G‐treated mice (0.21 ± 0.16 *versus* 3.16 ± 0.47; *P* < 0.0001). This anti‐arthritic effect of GF9 was comparable to that of 0.1 mg/kg dexamethasone (Dex) used as a positive control (data not shown). The effect is dose dependent as no therapeutic activity was observed for free GF9 administered at 2.5 mg/kg (data not shown).

To test whether incorporation of GF9 into macrophage‐targeted HDL increases its therapeutic efficacy in CIA, GF9‐dHDL and GF9‐sHDL were administered daily at 2.5 mg of GF9/kg. Despite a 10‐fold decrease in administration dose of GF9, the observed therapeutic effect of GF9‐HDL of either discoidal or spherical shape was comparable to that observed for free GF9 (Fig. 3A). Treatment with either GA/E31‐dHDL or GA/E31‐sHDL also reduced CIA severity with activity of GA/E31‐sHDL comparable to that observed for free GF9 at a higher dose (Fig. 3A). Together with our confocal data, these findings, for the first time, demonstrate that one peptide sequence can: 1) promote the self‐assembly of HDL; 2) target these particles to macrophages; and 3) provide therapeutic efficacy *in vivo*.

Interestingly, in contrast to vehicle‐ or GF9‐G‐treated mice with CIA, administration of any of the GF9 sequence‐containing formulations resulted in an increase in body weight comparable to that observed for non‐arthritic naïve mice (Fig. 3D).

In summary, these data collectively indicate that GF9 generates a strong anti‐arthritic effect in CIA, thereby providing the first experimental *in vivo* evidence of previously predicted immunomodulatory activity of this peptide in TREM‐1‐mediated autoimmune diseases including RA 18. Incorporation of TREM‐1 inhibitory GF9 sequences into macrophage‐specific HDL substantially increases their therapeutic efficacy probably because of targeted delivery and/or the prolonged circulatory half‐life of the peptide afforded by this strategy.

### Protection against cartilage damage and bone erosion in CIA

**Figure 4:**
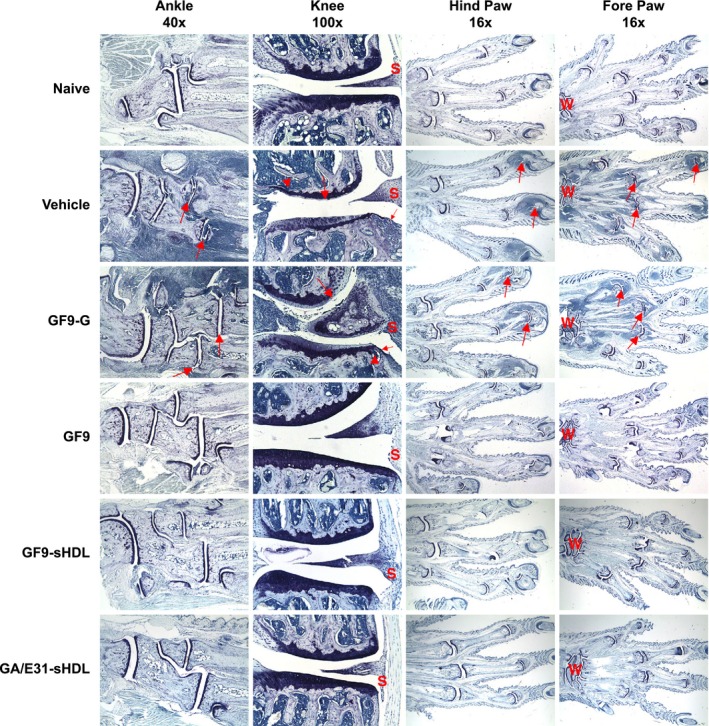
Treatment with TREM‐1 inhibitory GF9 sequences prevents pathological appearances from collagen‐induced arthritis (CIA). As described in the ‘Materials and Methods’, toluidine blue staining of the joints from mice with CIA treated with TREM‐1 inhibitory GF9 sequences or control peptide GF9‐G was performed. Photomicrographs of fore paws, hind paws, knees and ankles from representative mice are shown for each treatment group. For paws (original magnification × 16) and ankles (original magnification × 40), arrows identify affected joints. For knees (original magnification × 100), large arrow identifies cartilage damage, small arrow identifies pannus and arrowhead identifies bone resorption. W, wrist; S, synovium.

To determine whether treatment of mice with CIA using free and HDL‐bound TREM‐1 inhibitory GF9 sequences reduces chronic inflammation of synovial tissue, pannus formation, cartilage destruction and bone erosion, we next examined the histopathology of six joints from each animal including fore paws, hind paws and knees (Figs. 3 and 4).

No lesions were observed in non‐arthritic naive mice or in arthritic mice treated with Dex at 0.1 mg/kg (not shown). Vehicle‐treated arthritic mice had histopathology changes, consistent with those seen in type II CIA (Figs. 3 and 4). Microscopic alteration included infiltration of synovium and periarticular tissue with neutrophils and mononuclear inflammatory cells (inflammation), marginal zone pannus and bone resorption and cartilage damage (proteoglycan loss, chondrocyte death and collagen matrix destruction). Arthritic mice treated with GF9‐G at 25 mg/kg (Fig. 3B) or free GF9 at 2.5 mg/kg (data not shown) had histopathology parameters that did not differ significantly from vehicle‐treated mice. In contrast, all six‐joint mean histopathology parameters were overall significantly reduced in mice with CIA treated using either a higher dose of free GF9 or TREM‐1 inhibitory GF9 sequences incorporated into HDL (Fig. 3B). In line with our clinical observations (Fig. 3A), in mice treated with free GF9 at 25 mg/kg, a 96% reduction of summed scores compared to vehicle‐treated mice was observed, which was similar to that observed in Dex‐treated mice (99% reduction, data not shown). When compared to GF9‐G‐treated mice, mice treated with GF9 at the same dose had significantly reduced inflammation (94% reduction), pannus formation (98%), cartilage damage (96%), bone resorption (99%), periosteal bone formation (98%) and summed scores (97%) (Fig. 3C). A similar tendency was observed in mice treated with HDL‐based formulations (Figs. 3B and 3C). Reduction of histopathological scores observed in mice treated with GF9‐HDL and GA/E31‐HDL when compared to vehicle‐ or GF9‐G‐treated mice was in line with the clinical findings (Fig. 3A) and varied from 66 to up to 88% (Fig. 3B) depending on particle shape and composition.

Figure 4 shows representative photomicrographs of joints from arthritic mice treated with GF9 and GF9‐G in free form at the same dose of 25 mg/kg as well as with GF9‐HDL and GA/E31‐HDL. No differences were observed between arthritic mice treated with vehicle or control peptide GF9‐G (Fig. 4): the fore and hind paw joints had moderate to severe inflammation and cartilage damage with moderate pannus and bone resorption, as well as mild periosteal bone formation, in all joints. The ankle and knee joints from these mice had marked inflammation and moderate cartilage damage with pannus formation, bone resorption and periosteal bone formation (Fig. 4). Markedly thickened synovial membrane and capsule were observed in the vehicle‐ and GF9‐G‐treated mice as a result of pannus formation and inflammatory cell infiltration. The chronic inflammation destroyed the joint lining, including the cartilage and other nearby supporting structures, such as bone (Fig. 4). The formation of pannus probably resulted from overgrowth of the synoviocytes and the observed accumulation of inflammatory cells that led to deformed cartilage and bone. These findings agree with the observed clinical scores. In contrast, in the fore and hind paw joints of mice with CIA treated with free GF9 at 25 mg/kg, no or very minimal inflammation and minimal cartilage damage were observed (Fig. 4). The knee and ankle joints of these animals had no lesions or very minimal inflammation and only mild evidence of synovial membrane thickening with pannus formation, which falls within normal limits and is comparable to non‐arthritic naive mice (Fig. 4). Similar histopathology was observed in mice with CIA treated with either sHDL‐bound GF9 or sHDL‐bound GA/E31 (Fig. 4).

Thus, in line with clinical observations, histopathology findings demonstrated a specific protective effect of TREM‐1 inhibitory GF9 sequences against cartilage and bone erosion in CIA.

### Inhibition of cytokines in CIA

**Figure 5:**
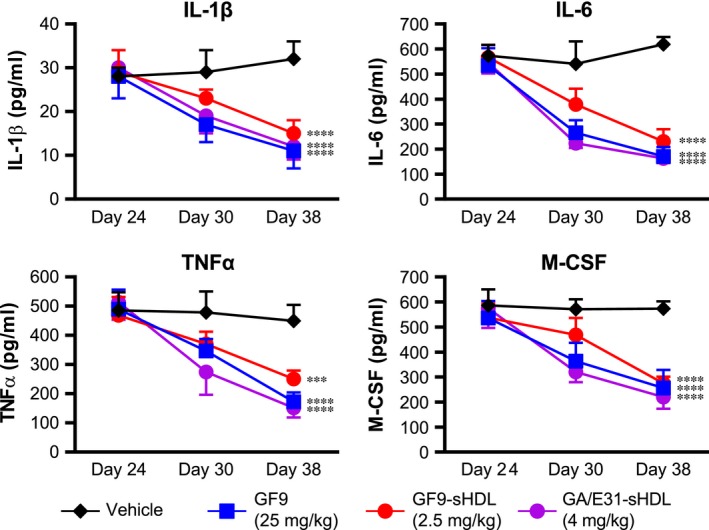
Treatment with TREM‐1 inhibitory peptide GF9 sequences reduces plasma cytokines in collagen‐induced arthritis. Plasma was collected on days 24, 30 and 38 from arthritic mice treated with vehicle (black), GF9 (blue), GF9‐loaded spherical HDL (GF9‐sHDL, red) and sHDL that contain an equimolar mixture of GA31 and GE31 (GA/E31‐sHDL, purple). Plasma samples were analysed for concentrations of interleukin‐1β (IL‐1β), IL‐6, tumour necrosis factor‐α (TNFα) and macrophage colony‐stimulating factor (M‐CSF). Results are expressed as the mean ± SEM (n = 5 mice per group). ***P <* *0.001; ****P <* *0.0001 *versus* vehicle.

The contribution of macrophage‐derived pro‐inflammatory cytokines and M‐CSF in CIA is well established 27, 28, and TREM‐1 activation is known to enhance the release of these cytokines 10, 18, 29. To further elucidate the molecular mechanisms underlying the reduction in severity of CIA in mice treated with TREM‐1 inhibitory GF9 sequences, we next analysed the plasma cytokine levels on day 24 at the start of treatment, on day 30 at around the disease peak and on day 38 when the arthritis swelling was at the plateau region. As expected, on day 24, no significant differences in cytokine levels between all the groups of mice with CIA were observed (Fig. 5). On days 30 and 38, no substantial changes in cytokine levels as compared to day 24 were observed in vehicle‐treated mice (Fig. 5) and in animals treated with GF9 at 2.5 mg/kg or GF9‐G at 25 mg/kg (data not shown). In contrast, in arthritic mice treated with GF9 at 25 mg/kg as well as in those treated with GF9‐HDL or GA/E31‐HDL, plasma levels of TNFα, IL‐1β, IL‐6 and M‐CSF were decreased on day 30 and more pronouncedly on day 38 (Fig. 5; only GF9, GF9‐sHDL and GA/E31‐sHDL are shown for illustrative purposes).

Thus, free and HDL‐bound TREM‐1 inhibitory GF9 sequences are effective in reducing the plasma cytokines TNFα, IL‐1β, IL‐6 and M‐CSF in a dose‐dependent manner.

## Discussion

The availability of new biologic therapies that target cytokines has greatly improved the management of RA 12. However, the response of patients with RA to cytokine blockers is often inadequate. For example, up to 30% of patients do not respond to TNF blockers 30. The use of cytokine blockers may also cause deleterious side effects such as serious infections, malignancies and septic arthritis 31, 32, 33.

Mechanistically, most of these agents bind free cytokines. Considering a high interindividual variability in cytokine levels in RA patients 34, this suggests that the therapeutic response to cytokine blockers as well as the risk for complications can depend on baseline cytokine levels. Indeed, in patients with RA, baseline TNF is associated with the clinical response to infliximab (TNF blocker): a higher dose is necessary in patients with a high baseline TNF, whereas lower doses of infliximab are sufficient for those with a low baseline TNF 35, 36. Further, for TNF blockers, an increased risk of malignancies is dose‐dependent 37. This leads to the critical need for personalization of treatment using cytokine blockers as well as for substantial efforts to avoid excessive immunosuppression. In addition, cytokine blockers do not protect against cartilage damage as effectively as they do against bone erosion 38.

TREM‐1 contributes to the pathogenesis of RA and may serve as a promising therapeutic target in RA to suppress the specific inflammatory response while preserving the immune system's ability to fight off infections 9, 10, 11, 13, 18, 29. In this study, we applied our technologies of ligand‐independent TREM‐1 inhibition and macrophage‐targeted drug delivery (Figs. 1B and 1C) to the CIA model of RA. As predicted 18, free and HDL‐bound TREM‐1 inhibitory GF9 sequences that employ SCHOOL mechanisms of action (Fig. 1B) are, for the first time, shown to ameliorate CIA and protect mice against cartilage and bone damage. In accordance with the SCHOOL model 16, 17, a control peptide GF9‐G with lysine replaced by glycine did not exhibit any therapeutic activity in CIA (Figs. 3 and 4), suggesting specificity for the observed therapeutic effect.

Mechanistically, similar to other SCHOOL peptides 18, 39, 40, 41, free GF9 self‐inserts into the plasma membrane from outside the cell and disconnects TREM‐1 from DAP‐12 (Fig. 1B, Route 1). The observed colocalization of GF9 and TREM‐1 in the membrane of macrophages incubated with free GF9 (data not shown) confirms this mechanism and is in line with the findings reported for other SCHOOL peptides 19, 21, 42. Together with the intriguing ability of different viruses to use the SCHOOL mechanisms to disarm TCR and other receptors involved in the host immune response 19, 24, these findings support our unifying hypothesis 19, 24, 43 that viral immune evasion strategies developed and optimized during millions of years of evolution of virus–host interactions can be practically used for the rational drug design of novel mechanism‐based therapies.

Considering the very short half‐life of peptides *in vivo* and the lack of cell specificity of the self‐insertion process, we used our reconstituted (synthetic) HDL‐based delivery platform to provide targeted delivery of GF9 to macrophages and to prolong the peptide half‐life (Fig. 1C) 18, 19, 22. As expected, in contrast to free GF9, which is inactive at 2.5 mg/kg, HDL‐bound GF9 at the same dose was therapeutically effective in treating mice with CIA (Figs. 3 and 4). Although the underlying molecular mechanisms of this phenomenon need to be further elucidated, one can suggest that this may result from targeted delivery of TREM‐1 inhibitory GF9 sequence to macrophages and/or from the prolonged circulatory half‐life of HDL‐bound peptides. Confocal data (Fig. 2) suggest that in line with our previous data 18, 22, 25, methionine‐sulfoxidized apo A‐I epitopes involved in the interaction of HDL with macrophages are exposed in all types of these HDL particles and provide intracellular delivery of TREM‐1 inhibitory GF9 sequences to macrophages, most probably in a receptor‐mediated manner. On the other hand, while the *in vivo* peptide half‐life is typically a few minutes 44, native dHDL and sHDL are characterized by much longer half‐lives up to 12‐20 hours and 3‐5 days, respectively 45, 46. This could result in less frequent administration, which in turn might improve compliance. Further, our confocal data *in vitro* (Fig. 2) and the therapeutic efficacy of HDL‐based formulations observed *in vivo* suggest that TREM‐1 inhibitory GF9 sequences intracellularly delivered to macrophages can reach their site of action in the membrane from inside the cell. The use of HDL as a vehicle for systemic delivery of drugs is increasingly important 47. Together with advanced clinical development of reconstituted HDL *per se* as a drug 48, the capability of synthetic apo A‐I peptides to functionally replace human apo A‐I demonstrated in mice with CIA (this study) as well as in mice with sepsis, atherosclerosis and lung cancer 18, 19, 22 encourages further development of this HDL‐based delivery platform.

To our knowledge, this study is first to demonstrate that TREM‐1 inhibitory functionality can be combined in one sequence with seemingly unrelated functionalities of other peptides resulting in multifunctional peptides. In the present study, trifunctional peptides GE31 and GA31 were shown to assist in the self‐assembly of HDL, target these particles to macrophages and inhibit TREM‐1. This suggests that multifunctional peptides that target TREM‐1 and/or other receptors expressed on macrophages can be designed and used in developing the next‐generation peptide‐based therapies for RA and other diseases.

At the molecular level, TREM‐1 activation mediates the production of multiple cytokines and growth factors that are involved in the pathogenesis of RA 49. Among them, TNFα, IL‐6 and IL‐1 serve as targets of cytokine‐blocking therapies that are currently in development (*e.g.,* IL‐21, IL‐23 and IL‐33), at different phases of clinical trials (*e.g.,* IL‐7, IL‐15, IL‐17 and M‐CSF), or approved (*e.g.,* TNFα, IL‐6 and IL‐1 blockers) 50. In the present study, TREM‐1 inhibitory GF9 sequences significantly reduced plasma levels of pro‐inflammatory cytokines and M‐CSF in mice with CIA in a specific and dose‐dependent manner. Interestingly, M‐CSF plays a role in promoting tumour growth and progression to metastasis 51. Currently, several inhibitors of M‐CSF or its receptor are in various stages of clinical development for cancer therapy 52. Thus, M‐CSF may represent an important mechanistic link between the anti‐arthritic activity of TREM‐1 inhibitory GF9 sequences observed in this study and the anticancer activity of GF9 previously observed in two xenograft models of NSCLC 18. This is in line with recent studies 53, where M‐CSF was suggested as the underlying factor facilitating tumour progression and metastasis in arthritic PyV MT mice. Further studies are needed to confirm this hypothesis.

In summary, in this study, we provide compelling *in vivo* evidence in support of the potent therapeutic effect of TREM‐1 inhibitory GF9 sequences in CIA. This further expands the range of applications for TREM‐1 SCHOOL peptide sequence‐based formulations from oncology and sepsis 18 to autoimmune diseases such as rheumatoid arthritis, systemic lupus erythematosus, colitis and inflammatory bowel disease, where blockade of TREM‐1 is of clinical importance. Together with our previous studies 18, 22, 25, this work also suggests potential theranostic applications of the HDL‐based delivery platform in autoimmune diseases.

## Conflicts of interest

Shen and Sigalov are employees of SignaBlok, Inc. No non‐financial conflicts of interest exist for any of the authors with respect to this study.

## Author contribution

ABS conceived and designed the experiments; ZTS and ABS performed the experiments; ZTS and ABS analysed the data; ZTS and ABS wrote the paper. All authors involved in drafting the article or revising it critically for important intellectual content, and all authors approved the final version to be published.
